# Evaluation of Highway Hydroplaning Risk Based on 3D Laser Scanning and Water-Film Thickness Estimation

**DOI:** 10.3390/ijerph19137699

**Published:** 2022-06-23

**Authors:** Wenchen Yang, Bijiang Tian, Yuwei Fang, Difei Wu, Linyi Zhou, Juewei Cai

**Affiliations:** 1 National Engineering Laboratory for Surface Transportation Weather Impacts Prevention, Broadvision Engineering Consultants Co., Ltd., Kunming 650200, China; tongjiywc@163.com (W.Y.); yuwei_fang@hotmail.com (Y.F.); 2 Yunnan Key Laboratory of Digital Communications, Kunming 650103, China; 3 Key Laboratory of Transportation Meteorology, China Meteorological Administration, Nanjing Joint Institute for Atmospheric Sciences, Nanjing 210008, China; zhoulinyi@cma.gov.cn; 4 Key Laboratory of Road and Traffic Engineering, Ministry of Education, Tongji University, Shanghai 201804, China; 1994wudifei@tongji.edu.cn (D.W.); jueweicai@126.com (J.C.)

**Keywords:** hydroplaning risk, water-film thickness, 3D laser scanning, LiDAR, pavement profile

## Abstract

Hydroplaning risk evaluation plays a pivotal role in highway safety management. It is also an important component in the intelligent transportation system (ITS) ensuring human driving safety. Water-film is the widely accepted vital factor resulting in hydroplaning and thus continuously gained researchers’ attention in recent years. This paper provides a new framework to evaluate the hydroplaning potential based on emerging 3D laser scanning technology and water-film thickness estimation. The 3D information of the road surface was captured using a vehicle-mounted Light Detection and Ranging (LiDAR) system and then processed by a wavelet-based filter to remove the redundant information (surrounding environment: trees, buildings, and vehicles). Then, the water film thickness on the given road surface was estimated based on a proposed numerical algorithm developed by the two-dimensional depth-averaged Shallow Water Equations (2DDA-SWE). The effect of the road surface geometry was also investigated based on several field test data in Shanghai, China, in January 2021. The results indicated that the water-film is more likely to appear on the rutting tracks and the pavement with local unevenness. Based on the estimated water-film, the hydroplaning speeds were then estimated to represent the hydroplaning risk of asphalt pavement in rainy weather. The proposed method provides new insights into the water-film estimation, which can help drivers make effective decisions to maintain safe driving.

## 1. Introduction

Hydroplaning is a significant factor affecting driving safety on highways and expressways [1]. According to the FWHA (Federal Highway Administration) report, 12.6% percent of the total accidents in 2000~2009 in the United States occurred on wet pavement due to hydroplaning resulting from low skid resistance [2]. The hydroplaning phenomenon is becoming an important safety issue in ITS and one of the most important sources of risk [3]. Investigation of the hydroplaning risk evaluation and prevention methods is necessary to improve traffic safety and prevent traffic accidents in emerging ITS.

Hydroplaning is a situation in which a vehicle tire rides up on a thin surface of the water, losing contact with the road surface and resulting in a sudden loss of control [4]. It has been a matter of concern for drivers on wet roads [5]. In the past decades, many experimental and numerical studies have been conducted to evaluate and predict hydroplaning risk [6,7,8]. It is widely accepted that the key factor resulting in hydroplaning is the water-film on the pavement [8]. A layer of water-film between tire and pavement would generate uplift force or pressure and then raise a portion of the tire off the pavement [9]. When vehicle speed exceeds a critical value, the tire is only supported by the water-film and loses all contact with the pavement [10]. The critical speed, named hydroplaning speed, has been widely studied to evaluate the hydroplaning risk for road safety management [6].

Water-film thickness is related to pavement texture, road slopes, and rainfall intensity. Pavement texture affects the water accumulation and dispersion of road surfaces. It determines the flow paths of water and a well-textured pavement can provide flow paths to allow water in front of the tire to be forced under pressure. Pavement slopes (cross and longitudinal slopes) also determine water flow paths. A road surface with appropriate slopes with no ruts or potholes is crucial to guarantee good drainage performance. In the past decades, many methods were proposed for water-film thickness estimation. Empirical and analytical methods are the two most common-used methods. Empirical methods were based on experiments and measured empirical data. One of the common-used empirical methods is using in-pavement or roadside liquid-film sensors. Plenty of sensors were developed since 1991. In recent years, the emerging optical fiber sensor technology is becoming the research focus due to its advantages of real-time high-precision and long-term monitoring [11,12,13]. However, it is still challenging to estimate large-scale water-film thickness because the optical fiber sensor can only measure the thickness at single points. Compared with the empirical method, the analytical method estimates the water-film thickness utilizing mathematical hydrodynamics models. The main inputs of the mathematical model are the rainfall intensity and road surface profiles. By capturing the profile information of the road surface, we can estimate the water-film thickness in different conditions of rainfall intensity.

The development of 3D laser scanning technology provides a new solution for measuring the road surface profile [14], and thus makes it possible for large-scale water-film thickness estimation. Table 1 compared the specifications of the three different water-film detection methods (in-pavement sensor, roadside sensor, and 3D laser scanning). As one of the most emerging technologies, the light detection and ranging (LiDAR) system can measure the 3D profile of the road surface and even the macro pavement texture [15]. Compared with the conventional laser scanning method, the LiDAR system can capture the laser point data of roads with a larger coverage (over 40 m-wide, which can cover all the lanes) and higher speed (>60 km/h). The captured laser point data of road contains rich information about road surface, including the geometrical features of interest (e.g., cracks and bumps), the pavement roughness, and even the skid resistance [16,17,18]. Although this technique is slightly less precise than other methods, it provides a rapid, large-scale solution for water layer estimation on roads. Since the laser point data includes the geometric information, we can estimate the water-film thickness by introducing the analytical models. Using this method, researchers [19,20] proved the feasibility of the 3D surface data for water-film estimation, demonstrating the feasibility of hydroplaning risk evaluation. However, current studies focus mainly on a relatively small region (one single lane) due to the coverage limits of the laser detection system. Most of them captured the road surface data based on the digital highway data vehicle equipped with two laser 3D cameras. The range of the two cameras can only cover one single lane but hardly measure the geometry information of the whole road surface, which cannot meet the real-time perception requirement for traffic safety. In order to ensure driving safety and provide in-time hydroplaning risk information, it is necessary to estimate the road surface condition rapidly and in a wide range. To this end, this study attempted to use the emerging LiDAR technology to rapidly capture the wide-range road surface geometry data and propose an algorithm to estimate the two-dimensional distribution of hydroplaning risks accurately, becoming an important module of the safety information service in future ITS.

**Figure 1:**
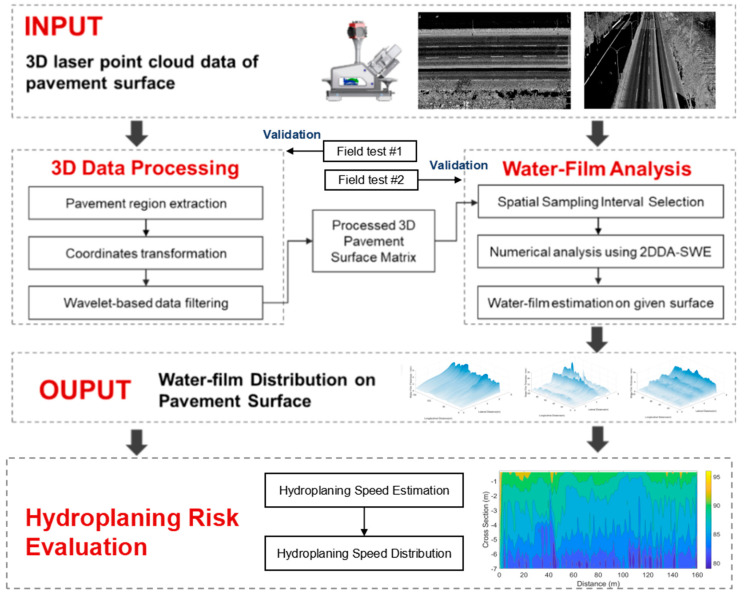
Pipeline of the proposed method.

The pipeline of the proposed method is illustrated in Figure 1. A vehicle-mounted LiDAR system firstly captures the 3D profile of the road. Then, the raw 3D point cloud data is processed to extract the road surface through coordination transformation and wavelet-based processing. Using the processed road surface data as the input, a numerical model of the two-dimensional depth-averaged Shallow Water Equations (2DDA-SWE) is adopted to solve the water-film distribution under different rainfall intensities. The 3D profile measurement and two-dimensional water-film thickness estimation are respectively validated through field tests. The effects of spatial sampling interval, the rainfall intensity, and the surface profiles of pavement sections (cross slopes\longitudinal slopes\rutting\rough surface) are investigated. The hydroplaning risk is then analyzed based on hydroplaning speed estimation based on the estimated water-film distribution data. The following sections describe the data and algorithms of each step in sufficient detail.

## 2. 3D Laser Scanning Data

LiDAR systems use a laser to measure the 3D information of the surrounding environment. The generic LiDAR systems include the stationary laser scanner, the airborne laser scanner, and the vehicle-mounted mobile scanning system. In this study, we used the vehicle-mounted mapping system to capture the LiDAR point cloud data of pavement. This mapping system integrates a multi-frequency GPS/GLONASS receiver, IMU, and a Riegl VUX-1HZ laser scanner, allowing us to measure the 3D road surface data under moving conditions. The performance of 3D laser scanning depends on the selection of laser channels, the vertical field of view, and the vertical resolution of laser beams. We installed the LiDAR sensor on the top of the test vehicle with no inclination to guarantee adequate coverage and maintain high accuracy. The height of the sensor was 6 ft above the ground. The scanning frequency, angular resolution, and scanning radius were set to be 75 Hz, 0.5°, and over 50 m, respectively. Under a fixed scanning frequency and radius, the density of the LiDAR point cloud is only related to the speed. A lower speed is always recommended to obtain a denser point cloud data, and thus the vehicle speed is controlled at about 40 km/h in this study, corresponding to the spatial interval of 0.5~2 cm.

### 2.1. Data Description

The output point cloud data of the LiDAR system include the information of 3D position (x, y, z), intensity, RGB, time, and heading angle. This study utilized the 3D position information to generate the 3D surface of the pavement and estimate the water-film distribution. As shown in Figure 1, the geometries of pavement and the surrounding environment can be effectively sensed by the LiDAR system.

### 2.2. 3D Data Processing

The raw laser point cloud data must be well-processed before water-film thickness estimation to be a standard input matrix for water-film analysis. To this end, we proposed a multi-step data processing method. The first step is to extract the valuable part (the point clouds of pavement) from the complex raw point cloud data. Then, a coordinate transformation algorithm was proposed to transform the global coordinates system into a local system. Finally, a wavelet-based filter was developed to capture the road surface’s basic geometry.

**Figure 2:**
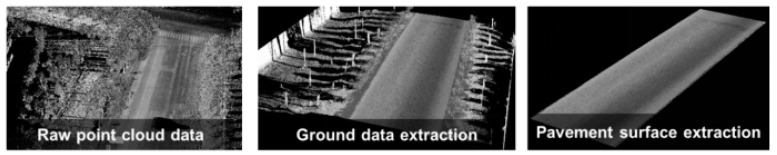
Steps of the pavement region extraction.

(1) Pavement region extraction (Figure 2).

The raw laser point cloud data contains plenty of redundant information, including the roadside buildings, the trees, the traffic lights, and even the moving vehicles, making the raw data too complex to process. As shown in Figure 2, the point clouds of the surrounding environment are inevitably measured when the LiDAR system is working. The point clouds of trees, buildings, and even the sidewalks are both redundant data for water-film thickness analysis. To extract the pavement point clouds data from the raw data, we firstly adopted a passthrough filter to separate the ground part from the others. The threshold was determined based on the observed height value of the pavement.

Although the passthrough filter eliminates the most point clouds of trees, lights, and buildings, some outliers with low heights can hardly be obliterated. Therefore, we then used the random sample consensus (RANSAC) method to extract the pavement region from the ground point clouds.

**Figure 3:**
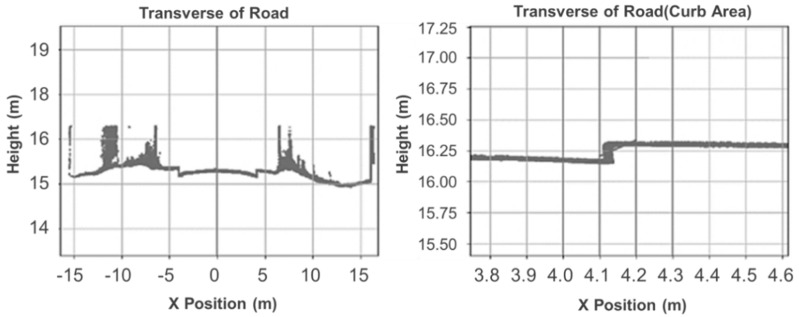
Height-based method for pavement region separation, proposed by Wei [21].

The last step for pavement region extraction is to identify the curbs and determine the boundaries of the pavement region. The curbs are located between the sidewalks/central strips and the pavement. The point clouds of curbs can easily be identified based on the height differences, which are generally 10~20 cm. Wei [21] proposed a height-based method for pavement region separation, as shown in Figure 3. The section is firstly divided into several segments along the transverse direction. In each segment, the least square method is used to fit the points and calculate the estimated slope of the plane. For the points in the curb region, the slopes would be relatively high compared with the pavement region. Then, the following equation is determined for curb identification:

(1) $$\{\begin{array}{ll} if\;S_{\min}<S<S_{\max} & curb\;candidate\\ else & non-curb\;region \end{array},$$

where *S* denotes the estimated slope of the segment, *S*_min_ and *S*_max_ denote the lower and upper thresholds. This method can extract the bidirectional information on the road surface for the roads without central strips. However, only one-direction surface information can be extracted for the roads with central strips.

(2) Coordinates transformation.

The raw point cloud data is measured using a global coordinates system instead of a local system, making it inappropriate for point cloud data meshing. Coordinate transformation is required before further analysis. Thus, the translation and rotation equations were adopted to process the raw point cloud data, as shown in Equations (2) and (3).

(2) $$\left\{\begin{array}{c} x_{t}\\ y_{t}\\ z_{t} \end{array}\right\}=\left\{\begin{array}{c} x\\ y\\ z \end{array}\right\}+T,T=-\frac{1}{n}\left\{\begin{array}{c} \overset{n}{\underset{i=1}{\sum }}x_{i}\\ \overset{n}{\underset{i=1}{\sum }}y_{i}\\ \overset{n}{\underset{i=1}{\sum }}z_{i} \end{array}\right\},$$

where the subscript *t* indicates the translated coordinates and *n* is the number of points in the pavement region. Then, the spatial coordinates are rotated by the following matrix operation:

(3) $$\left[\begin{array}{c} x_{r1}\;x_{r2}\cdots x_{rm}\\ y_{r1}\;y_{r2}\cdots y_{rm}\\ z_{r1}\;z_{r2}\cdots z_{rn} \end{array}\right]=R\cdot \left[\begin{array}{cccc} x_{t1} & x_{t2} & \cdots & x_{tn}\\ y_{t1} & y_{t2} & \cdots & y_{tn}\\ z_{t1} & z_{t2} & \cdots & z_{tn} \end{array}\right],R=\left[\begin{array}{ccc} \cos\theta & -\sin\theta & 0\\ \sin\theta & \cos\theta & 0\\ 0 & 0 & 1 \end{array}\right],$$

where *θ* denotes the direction angle of the road centerline. In this study, we proposed a linear-fitting-based algorithm for estimating the direction angle and calculating the rotated matrix, shown as follows (Algorithm 1):
**Algorithm 1. Numerical Algorithm for Coordinates Rotation****STEP 1.** Input the 3D surface data, and extract the plane coordinates *x*, *y*.**STEP 2.** Estimate the slope of the plane coordinates *s* using linear fitting.**STEP 3.** Verify the slope condition:(i) if *s* ≤ 0.05, proceed to Step 6.(ii) if *s* > 0.05, proceed to Step 4.**STEP 4.** Calculate *θ* based on *s* using the equation: θ=−1⋅actan(s)
**STEP 5.** Calculate the rotated matrix using Equation (3). Return to Step 2 and continue the slope calculation until the slope condition is satisfied.**STEP 6.** Output the final coordinates matrix as the rotated result.

**Figure 4:**
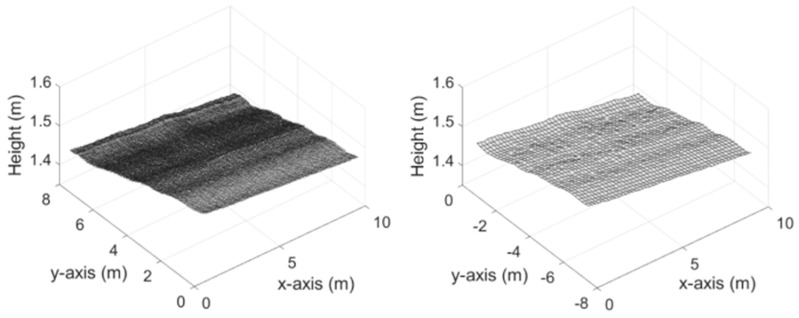
Coordinates transformation and surface meshing.

Once the coordinates were translated and rotated, a quadratic surface fitting method was used to mesh the discrete cloud points. Figure 4 shows the meshed result after coordinates transformation and surface fitting. The meshing size was set to be 0.1 m. The selection of meshing size was discussed in Section 3 based on the sampling interval for water-film thickness estimation.

(3) Point cloud data denoising and filtering.

The measured point cloud data usually contains abrupt data due to obstacles on pavement, such as leaves. The abrupt data appeared as prominent abrupt peaks in the 3D data graph. We proposed a wavelet-based method for 3D data filtering to remove such outliers.

A wavelet transform approach is a widely accepted tool for signal filtering and multi-scale analysis. Specifically, wavelet transform uses adjustable-width basis functions (called wavelets) to decompose a signal into an equivalent approximation signal and a detail signal. The approximation signal is subsequently decomposed into another set of approximation and detail signals. The decomposition step can be repeated several times to extract the low-frequency and high-frequency components.

By applying several wavelet transforms on the target signal, we can obtain the decomposition results in different scales, corresponding to different frequency bands. The obstacle-induced outliers in the point cloud data belong to the high-frequency information and thus can be separated through wavelet transform. Therefore, the 3D data can be well-filtered through wavelet transform by removing the high-frequency signals and reserving the low-frequency components.

**Figure 5:**
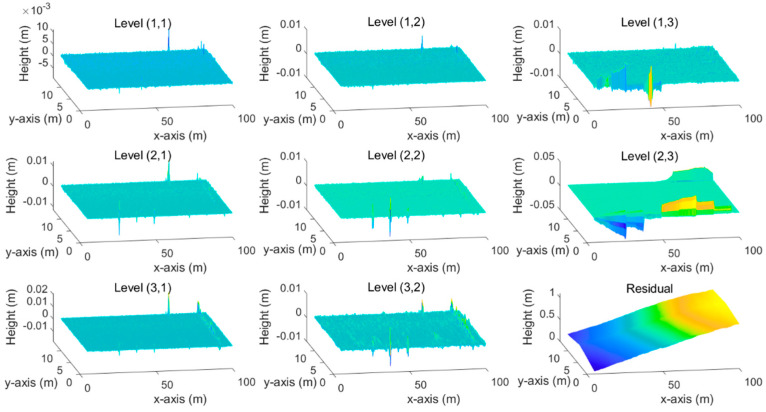
Wavelet decomposition results.

The selection of the wavelet basis (mother wavelet) is an essential factor affecting the wavelet decomposition performance. It should be as similar to the original signal to ensure a better decomposition result. DbN (N = 1, 2, ……, *n*) and symN (N = 1, 2, ……, *n*) are the most common-used mother wavelets for pavement profile analysis [22]. As Weng’s study [14] recommended, we selected the mother wavelet “sym4” for developing the wavelet-based filter. The level of wavelet decomposition determines the fineness of the decomposition result. This study utilized the MATLAB Wavelet Toolbox to decompose the raw data into three levels (in both horizontal and vertical directions), two of which were the detailed signals denoted as Level 1 (0.1~0.2 m), Level 2 (0.2~0.4 m). Level 3 (>0.4 m) was the residual signal. As we decomposed the raw 3D signal in horizontal and vertical directions, nine decomposed components were obtained, including eight detailed signals and one residual signal, as shown in Figure 5.

**Figure 6:**
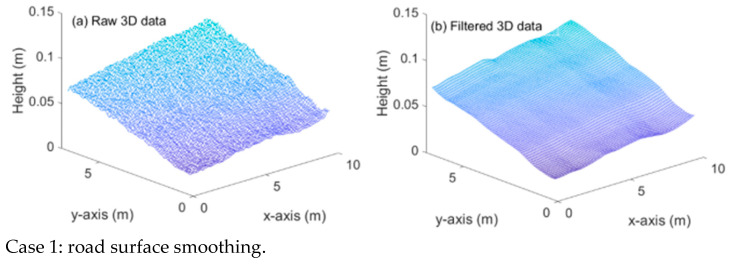

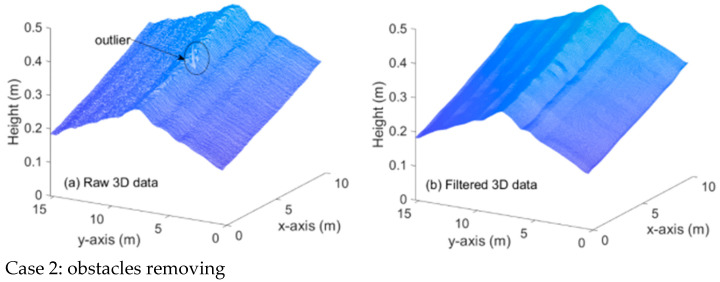
Raw 3D data and filtered 3D data of Case 1 (a,b) and Case 2 (a,b).

Figure 6 shows the raw data and filtered results. It is observed the 3D data is well-smoothed, which is more appropriate for water-film analysis. From Figure 6 we also see that the obstacle-induced outliers are eliminated through the proposed wavelet-based filtering, while the 3D profile of the road surface, including the rutting, is well reserved.

### 2.3. Validation for 3D Road Surface Measurement

**Figure 7:**
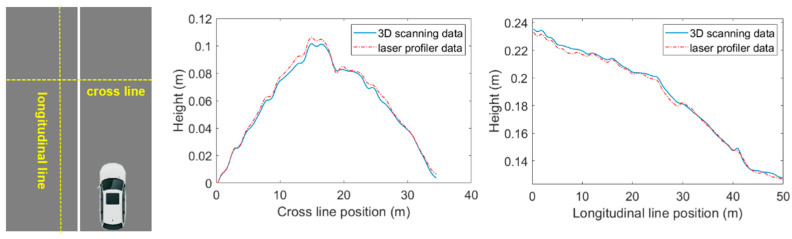
Validation for 3D road surface measurement.

The road surface measurement performance of the LiD technique is required to be validated to ensure the reliability of subsequent water-film thickness prediction. We introduced a portable laser profiler in the validation test, a common-used device for road profile measurement and pavement roughness evaluation. The validation tests were conducted on an asphalt highway in Shanghai, and a road section of 33 m × 50 m was selected for road elevation measurement comparison. Since the portable laser profiler can only measure the one-dimensional road profile, we set two test lines (see Figure 7: longitudinal line and cross line) for comparison.

The LiDAR system was first applied to measure the 3D surface of the selected road section, road profile data corresponding to the two test lines were then extracted from the 3D point cloud data. Then, we used the portable laser profiler to measure the road profiles of the two test lines. Figure 7 shows the comparison results between the 3D scanning and laser profiler data. The two elevation curves match well in both cross-line and longitudinal lines. The average differences were 1.7 mm and 2.1 mm, respectively, demonstrating that the Lidar-based 3D scanning can precisely measure the road surface profiles.

## 3. Water-Film Prediction Based on 3D Surface Data

### 3.1. Governing Equations

The water film accumulating on the road surface is much smaller than the horizontal dimension of the pavement area. Moreover, the vertical component of water velocity is negligible compared to the horizontal component. Thus, the distribution of water-film can be described using the two-dimensional depth-averaged Shallow Water Equations (2DDA-SWE) [23,24]. In conservative form, the 2DDA-SWE is written as the following equation:

(4) $$\frac{\partial U}{\partial t}+\frac{\partial F\left(U\right)}{\partial x}+\frac{\partial G\left(U\right)}{\partial y}=Q,$$

in which

(5) $$U=\left(\begin{array}{l} h\\ uh\\ vh \end{array}\right),F=\left(\begin{array}{l} uh\\ u^{2}h+\frac{1}{2}gh^{2}\\ uvh \end{array}\right),G=\left(\begin{array}{l} vh\\ uvh\\ v^{2}h+\frac{1}{2}gh^{2} \end{array}\right),\;Q=\left(\begin{array}{l} q_{r}\\ ghS_{0,x}-ghS_{f,x}\\ ghS_{0,y}-ghS_{f,y} \end{array}\right)$$

where *h* is the water-film thickness (m), *u* and *v* are the horizontal component of water velocity along *x* and *y* directions, respectively (m/s), *q_r_* is the rainfall intensity (m/s), *g* is gravity constant (m/s^2^), *S*_0_ is the bed slop which can be directly calculated with 3D surface data:

(6) $$S_{0,x}=-\frac{\partial z}{\partial x},S_{0,y}=-\frac{\partial z}{\partial y},$$

where *z* is the pavement elevation. *S_f_* is the friction term which is derived by Manning’s Equations as follows:

(7) $$S_{f,x}=\frac{n_{c}^{2}u\sqrt{u^{2}+v^{2}}}{h^{4/3}},S_{f,y}=\frac{n_{c}^{2}v\sqrt{u^{2}+v^{2}}}{h^{4/3}},$$

where *n_c_* is the Manning roughness coefficient.

### 3.2. Numerical Algorithms

The governing equations consist of three non-linear partial differential equations commonly solved by numerical methods. The finite volume method (FVM) and HLL approximate Riemann solver are implemented to obtain the solution of the 2DDA-SWE.

**Figure 8:**
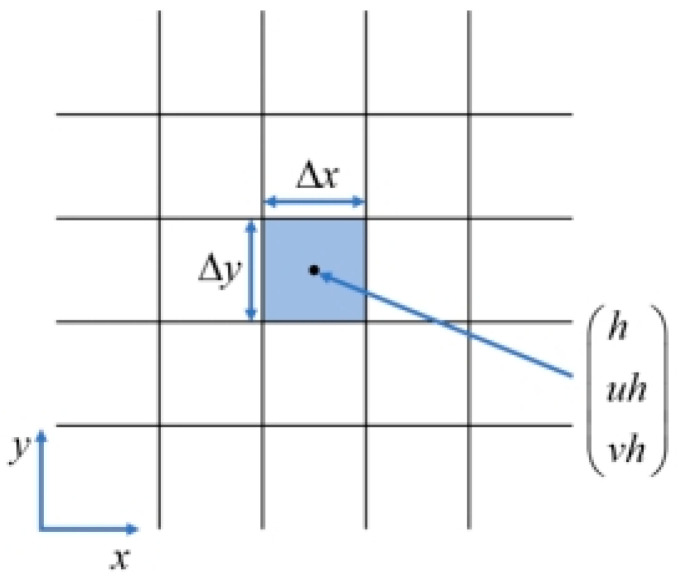
Spatial discretization of cell-centered finite volume scheme.

A cell-centered finite volume scheme based on a cartesian grid is used for spatial discretization. The averaged variables are stored at the center of the grid, and each grid is defined as the control volume. The two-dimensional cartesian grid is shown in Figure 8.

The time discretization is performed by using the integral conservation form of 2DDA-SWE, which can be obtained by integrating (1) over each computational cell Ω*_i_* with area Ai:

(8) $$\int_{\Omega_{i}}\frac{U}{\partial t}d\Omega +\oint_{\Gamma }E\cdot nd\Gamma =\int_{\Omega_{i}}Qd\Omega ,$$

where *n* = (*n_x_*, *n_y_*) denotes the outward unit normal vector of cell Ω*_i_* and Γ is the boundary of cell Ω*_i_*, *E =* (*F*, *G*) is the flux passing through the cell boundary. Using the TVD Runge-Kutta method of two-order accuracy for time discretization [25], the discretized form of Equation (8) can be written as:

(9a) $${\hat{U}}^{n+1}=U^{n}+\Delta t\left(Q^{n}-E^{n}\cdot n\frac{d\Gamma }{d\Omega }\right)$$

(9b) $$U^{n+1}=\frac{1}{2}U^{n}+\left[\frac{1}{2}{\hat{U}}^{n+1}+\frac{1}{2}\Delta t\left({\hat{Q}}^{n+1}-{\hat{E}}^{n+1}\cdot n\frac{d\Gamma }{d\Omega }\right)\right],$$

The calculation of intercell flux *E* is called the Riemann problem mathematically, which can be solved by Harten-Lax-van Leer (HLL) approximate Riemann solver [26,27]. Moreover, to ensure the stability of the numerical algorithm, the iteration time step is constrained by Courant-Friedrichs-Lewy (CFL) condition, which is defined as:

(10) $$CFL=\frac{\Delta t}{\min(\Delta x,\Delta y)}\left(\sqrt{u^{2}+v^{2}}+\sqrt{gh}\right)\leq 1,$$

The ghost grid method calculates the grid fluxes at the boundary. By adding ghost grids at the boundary, the interface fluxes of the original boundary grids can be calculated in the same way as the internal grid. The variable values of the ghost grids are determined by the pavement drainage condition. Since the water cannot be discharged freely across the grid for the boundary with curbs, the wall boundary conditions are applied. The ghost grid and boundary grid have the same water film thickness and opposite normal water velocity. For the free drainage boundary, the water can be discharged freely without the influence of the roadside facilities, and the open boundary conditions are applied. In this case, the water velocity of the ghost grid is the same as the boundary grid and the water film thickness is calculated by linear extrapolation.

Based on Equations (4)–(10), the numerical algorithm for calculating the spatial and temporal distribution of water-film is summarized as follows (Algorithm 2):
**Algorithm 2. Numerical Algorithm for 2DDA-SWE****STEP 1.** Input 3D surface data, rainfall intensity data, and initial parameters. The values of *z*, *q_r_*, *g,* and *n_c_* are known. Set the calculation period T and initial the model time *t* = 0.**STEP 2.** According to solution time and accuracy requirements, set time step Δ*t* and spatial step Δ*x* and Δ*y*.**STEP 3.** Calculate the intercell flux by Equations (7)–(9).**STEP 4.** Update the model time to *t* = (*n* + 1) Δ*t* by Equation (6).**STEP 5.** Verify the CFL condition:    (i) if CFL ≤ 1, proceed to Step 6.    (ii) if CFL > 1, increase Δ*x* and Δ*y* or decrease Δ*t*. Then return to Step 3.**STEP 6.** Return to Step 3 and continue until the calculation period is completed.

### 3.3. Model Parameter Acquisition

For model application, parameters such as rainfall intensity, Manning roughness coefficient, and pavement elevation must be obtained first. The rainfall intensity is calculated by the real-time cumulative precipitation. The relevant data can be collected from the rain gauge or meteorological station. The Manning roughness coefficient is determined by the pavement texture, which can be obtained by referring to relative standards or calculated based on the research of Stong and Reed [28].

**Figure 9:**
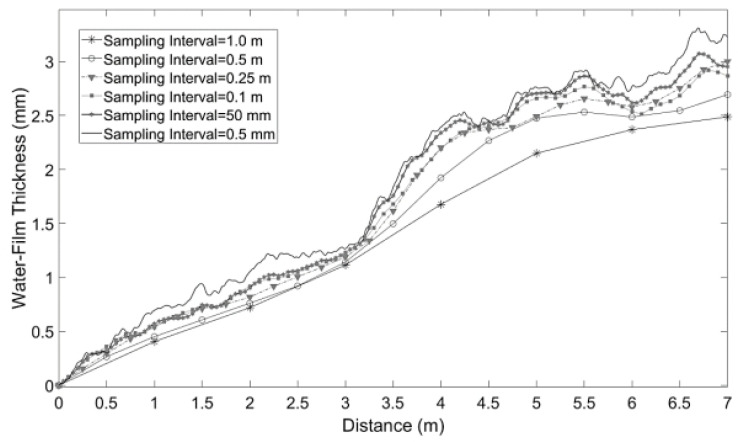
Water-film thickness estimation results using different sampling intervals.

The pavement elevation obtained from the 3D surface data is the critical influencing parameter of the water-film distribution. The sample interval of the data directly affects the calculation accuracy and efficiency. Acceptable sample interval leads to a more precise calculation result, but on the other hand, more time consumption will be generated. Therefore, to determine the optimal sample interval of 3D surface data, the water-film thickness distribution of the same pavement under different sample intervals was calculated by implementing the 2DDA-SWE model on a flat road surface. The results are shown in Figure 9 and Table 2. The calculated water-film thickness under the minimum sample interval (0.5 mm) is taken as the actual value. It is indicated that the calculated results are gradually close to the actual value with the decrease of the sample interval. When the sample interval is no more than 0.25 m, the difference between the calculated and actual values is within 0.3 mm, which satisfies the calculation requirements. However, the time consumption is significantly increased with the further decrease of the sample interval. A 500 times sample interval reduction will lead to more than 80,000 times the time consumption. As a result, after balancing the calculation accuracy and consumption, the sample interval in the range of 0.1 m to 0.25 m is selected as the optimal range.

### 3.4. Model Validation

**Figure 10:**
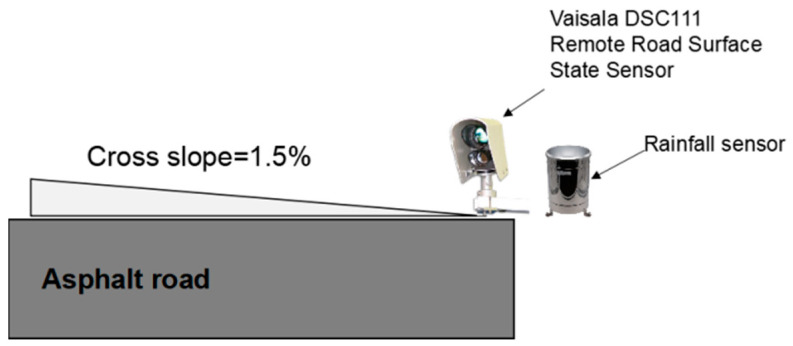
The schematic diagram of the validation test.

This part aims to validate the water-film thickness estimation algorithm on a given road surface. The model validation was conducted based on field monitoring data collected from a highway in Shanxi, China. Since the road section for validation was a new-constructed road, the road surface was regarded as a flat surface with longitudinal and cross slopes. The longitudinal and cross slopes were measured using a high-precision Total Station (TS): longitudinal slope—0.15%; cross slope—1.5%. A remote road surface state sensor was used to measure the water-film thickness on the road surface based on the spectroscopic measuring principle. Figure 10 shows the schematic diagram of the validation test. The sensor was installed on the roadside at the height of 35 cm, measuring the water-film thickness of the outer lane in real-time. A rainfall sensor was also installed nearby to capture the rainfall intensity information. Once the rainfall information was well-collected, the numerical method was applied to calculate the water-film thickness during the rainfall period.

**Figure 11:**
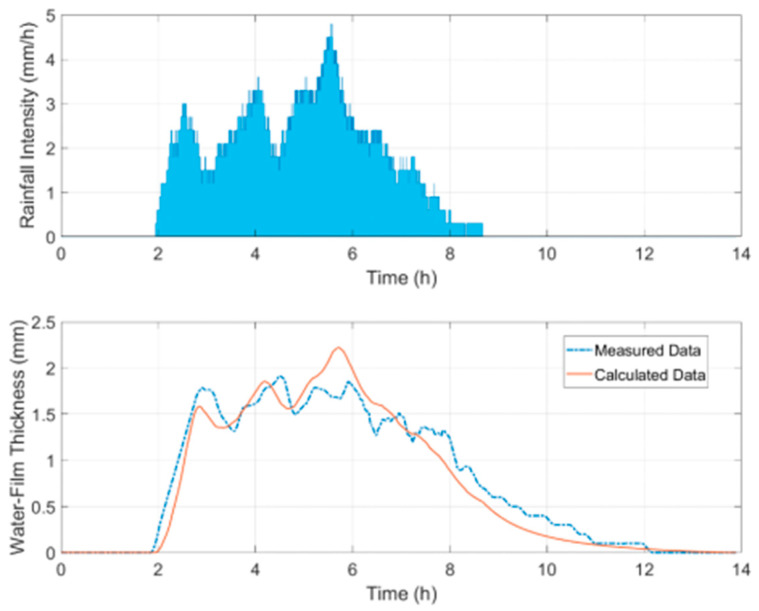
The validation result during a certain rainfall event.

During the numerical calculation, the road surface with the measured slopes was first developed, the rainfall intensity data was then put into the 2DDA-SWE-based model, and the time series of water-film thickness were then solved following the proposed procedures. Figure 11 shows the result comparison of the calculated water film and the measured data. It is first seen that the variation trend of water-film thickness with time was consistent with the rainfall curve. It is also noted that the difference between the calculated and measured data was relatively small, and the trend was consistent. The root mean square error and the maximum error of results are 0.17 mm and 0.76 mm, proving the model’s reliability in calculating water-film thickness distribution.

## 4. Water-Film Thickness Estimation on Road Surfaces with Different Profiles

**Figure 12:**
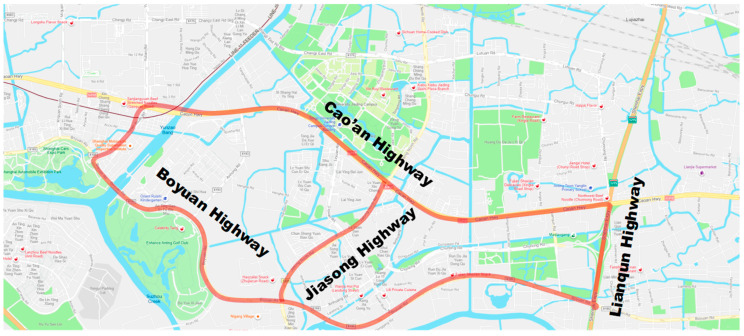
The tested highways.

The geometry of the road surface is an important factor affecting the drainage and water-film distribution on the road surface. Cross slopes, longitudinal slopes, ruts, and unevenness of the road surface will affect the drainage path on the pavement, resulting in uneven distribution of water film. To analyze the water-film distribution on road surfaces with different geometry types, we selected several samples of measured 3D surface data for water-film analysis based on the proposed algorithms. The sample data was measured on four highways in Shanghai, as shown in Figure 12. Three scenarios were considered: surface with slopes, surface with rutting, and roughness surface, as listed in Table 3. The pavement type of the four highways is impervious SMA (stone matrix asphalt); thus, the effect of water seepage was not considered. According to the historical data, the rainfall intensity was set to be 2.925 mm/min, which is the ten-year precipitation in Shanghai. The open boundary conditions are applied in this part of the analysis.

### 4.1. Surface with Slope

**Figure 13:**
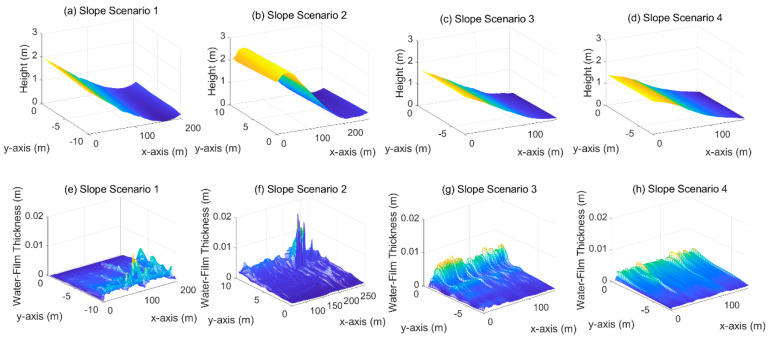
Water-film estimation results on road surfaces with a slope. (**a**–**d**): 3D road surface of slope scenario 1~4; (**e**–**h**): water-film thickness distribution of slope scenario 1~4.

Figure 13 shows the calculation results of water-film thickness on the road surfaces with slopes, which is 1–2% approximately. For the road surfaces with good roughness and no obvious rutting, the water-film thickness is mainly within 1 cm under heavy rain, indicating that the pavement drainage performance is good. It is also observed that cross slope has a more significant effect than longitudinal slope on the pavement drainage. The thick water film occurs at the curbs instead of the slope bottom. Moreover, note that the proposed water-film thickness estimation method did not consider the effect of roadside drainage wells, and thus the calculation results are inconsistent with the actual situation. In practical application, it can guide the design of drainage wells based on the positions of high-water-film-depth regions.

### 4.2. Surface with Rutting

**Figure 14:**
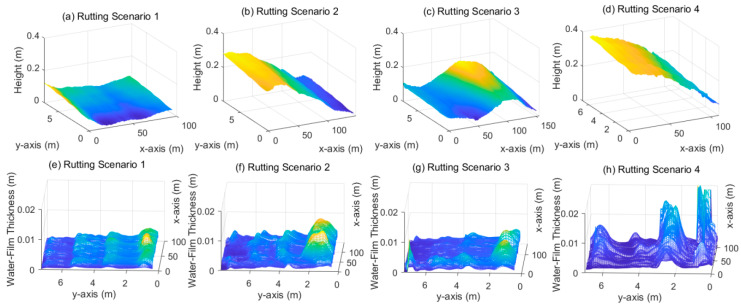
Water-film estimation results on road surfaces with rutting. (**a**–**d**): 3D road surface of rutting scenario 1~4; (**e**–**h**): water-film thickness distribution of rutting scenario 1~4.

Figure 14 shows the calculation results of water-film thickness on the road surfaces with rutting. It can be seen that the rutting affects the water-film distribution very significantly. The distribution of water-film thickness shows an apparent “double peak” shape, and the water-film thickness on the outer lane (usually the truck lane) is higher than that on the inner lane. The maximum water-film thickness can even reach over 2 cm. Moreover, there is no significant difference between the start and end regions, indicating that the impact of rutting is much more significant than that of the longitudinal road slope.

### 4.3. Rough Surface

**Figure 15:**
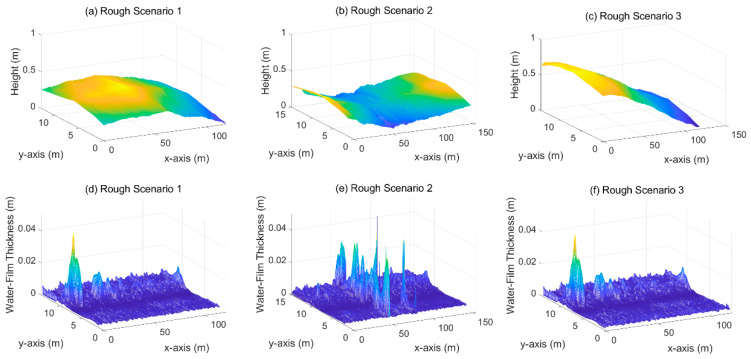
Water-film estimation results on rough road surfaces.(**a–c**): 3D road surface of rough scenario 1~3; (**d**–**f**): water-film thickness distribution of rough scenario 1~3.

Figure 15 shows the calculation results of water-film thickness on rough surfaces, the International Roughness Indexes of the three road sections are in the range of Scenario 1 in [2.59, 4.51], Scenario 2 in [4.22, 7.10], Scenario 3 in [3.52, 10.27]. The water-film distribution on a rough surface is irregular compared with slope and rutting scenarios. The local unevenness (depression, corrugation, and shoving) would change the water path on the road surface, resulting in thick water-film on those regions. As shown in Figure 13, the maximum water-film thicknesses in the three scenarios exceed 3 cm, much larger than in the other scenarios.

## 5. Hydroplaning Risk Evaluation

The hydroplaning speed is usually used to evaluate the hydroplaning risk by comparing it with the posted speed limit. Thus, the key to hydroplaning risk evaluation is to estimate the hydroplaning speed based on the road surface parameters. Various water-film-based methods have been developed to estimate hydroplaning speed based on analytical derivations or empirical formulas. It is widely accepted that the pavement hydroplaning speed is highly associated with water-film thickness, mean texture depth (MTD), and tire parameters (pressure and tread depth). Selecting an appropriate hydroplaning speed estimation model for different pavement scenarios is required.

**Figure 16:**
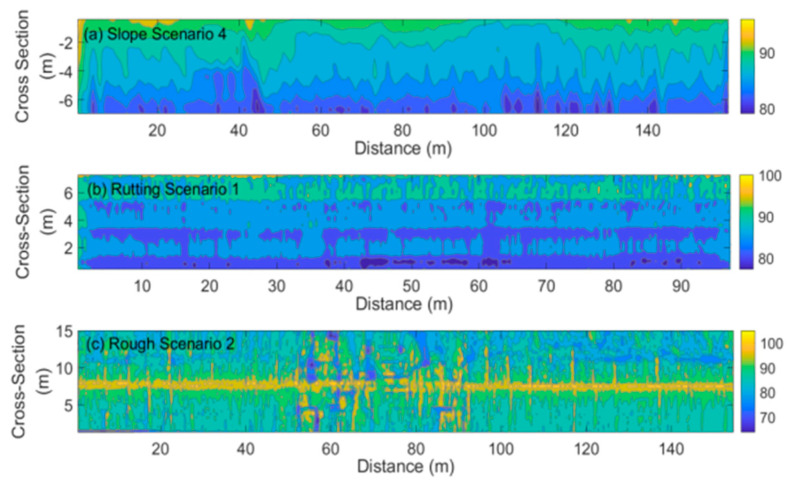
Hydroplaning Speed Estimation using the Gallaway model. (**a**). Hydroplaning speed distribution on the slope scenario 4; (**b**). Hydroplaning speed distribution on the rutting scenario 1; (**c**). Hydroplaning speed distribution on the rough scenario 2.

**Figure 17:**
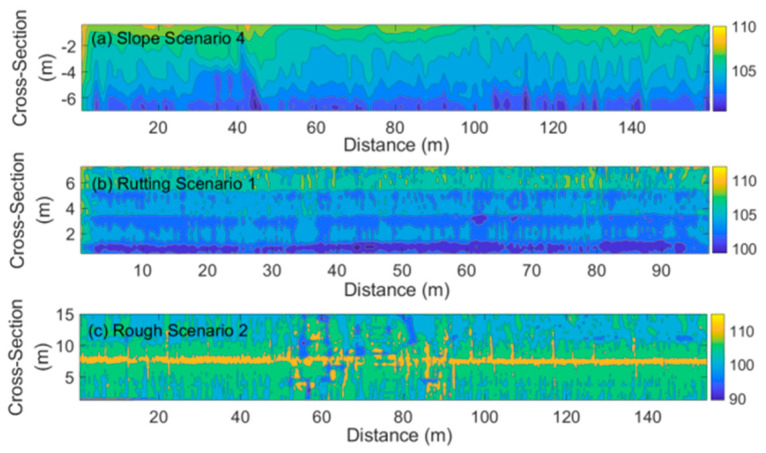
Hydroplaning Speed Estimation using the USF model. (**a**). Hydroplaning speed distribution on the slope scenario 4; (**b**). Hydroplaning speed distribution on the rutting scenario 1; (**c**). Hydroplaning speed distribution on the rough scenario 2.

In this study, we adopted the Gallaway model [29] and the USF (University of South Florida) model [30] based on Luo’s DFT (Dynamic Friction Tester) test results [20], which indicated that the two models provide more accurate results in hydroplaning speed prediction than other models. The Gallaway model is a classic model developed in 1979 by Gallaway B.M. et al. [29]. for the US Department of Transportation. It should be noted that the Gallway formula for hydroplaning speed estimation is only valid for speeds up to 55 km/h or 95 km/h, and it is recommended that a maximum water-film thickness of 4 mm should be achieved. The USF model was proposed by Ong and Fwa in 2007 [8]. It was developed by a comprehensive finite element model formulated to predict hydroplaning conditions accurately. This model considered the effect of wheel load, an important parameter affecting hydroplaning. The formulas of the two models are as follows (Table 4):

Where *SD* denotes the spin-down ratio (fixed as 1.0), *P_t_* denotes the tire’s inflation pressure, *TD* is the tire tread depth, *W* is the wheel load. For the selected sample road surfaces, we adopted the following variables (Table 5):

We can estimate the hydroplaning speed distribution based on the water-film distribution using the hydroplaning speed estimation models. Figure 16 (Gallaway model) and Figure 17 (USF model) illustrate the estimation results of three typical scenarios: Slope Scenario 4 in Figure 13, Rutting Scenario 1 in Figure 14, and Rough Scenario 2 in Figure 15. Note that the road surface of the Rough Scenario 2 was measured in two ways, and the yellow center area corresponds to the upper edge of the pavement. It is observed that the hydroplaning speed estimation results of the two models have apparent similarities in their distribution contours, although the calculated hydroplaning speed values have a maximum difference of 20 km/h. Moreover, it is observed that hydroplaning is more likely to occur on rutting tracks and uneven sections. The hydroplaning speed can be lower than 70~90 km/h, almost close to the highway’s speed limit. The vehicle should decelerate or steer to avoid hydroplaning in these regions.

## 6. Conclusions

Rapid detection and evaluation of highway hydroplaning risk has always been the focus of traffic safety research. As the core factor resulting in hydroplaning, the water-film on the road surface can significantly reduce the friction between tire and road surface, influencing public transportation health.

This paper proposes to develop a new rapid method for water-film thickness estimation on asphalt pavement based on 3D laser scanning, becoming an essential component in the ITS for improving driving safety and preventing traffic accidents. This method’s basic idea is to use a numerical method to estimate the water-film distribution based on LiDAR-captured 3D road surface data. Compared with traditional methods, this method allows us to rapidly predict the water-film thickness on a large scale (over 10 m-width, 100 m-length) within 10 s. The water-film estimation results were well-validated using the measured data on a highway in Shanxi, China.

Through applying this method to three different types of road surfaces on four highways, the results revealed that the pavement profile significantly impacts the water-film distribution on the pavement. For the road surface with slope, it is found that the water-film thickness becomes greater as the distance from the upper edge of the pavement increases. For the road surface with rutting, the water film on the rutting tracks is significantly thicker than in other regions. The maximum water-film thickness reached 2 cm, increasing the hydroplaning risk significantly. The water-film distribution on rough road surfaces shows an irregular pattern. The water film is much thicker in the depression region than in other regions. Using the Gallaway and USF models to calculate the hydroplaning speeds based on the estimated water-film thickness, it is found that the minimum hydroplaning speed on the rutting tracks and rough regions can be lower than 90 km/h, significantly affecting driving safety. Driving vehicles should avoid rutting tracks and rough regions on rainy days or take appropriate measures to prevent traffic accidents.

It should be emphasized that this study was focused on the hydroplaning risk evaluation method. We did not study how to improve driving safety on wet road surfaces. It would also be an interesting topic for further research. Our future work will focus mainly on the effect of rainfall intensity, and a detailed vehicle control strategy for safe driving in rainy weather will be studied.

## Figures and Tables

**Table 1:** Comparison between different water-film thickness measurement techniques.

Techniques	Rationale	Road Destructive	Measurement Range	Precision
In-pavement monitoring [11]	Directly measuring water-film thickness by the embedded sensor	Road destructive	Point measurement width < 10 cm	Resolution < 0.1 mm
Roadside detection [19]	Measuring water-film thickness by infrared remote sensing technology	Non-destructive	Point measurement width < 50 cm	Resolution < 0.1 mm
3D laser scanning [20]	Measuring the 3D profile of pavement and estimating the water-film thickness	Non-destructive	Continuous measurement width > 10 cm	Resolution < 0.3 mm

**Table 2:** Time Consumption for Different Sample Intervals.

Sample Interval	0.5 mm	50 mm	0.1 m	0.25 m	0.5 m	1 m
Time Consumption	83,134.02 s	11.02 s	3.26 s	1.01 s	0.78 s	0.67 s

**Table 3:** Scenarios for water-film thickness estimation.

Scenarios	Typical Geometry
Surface with slope: four samples Cao’an Highway Boyuan Highway	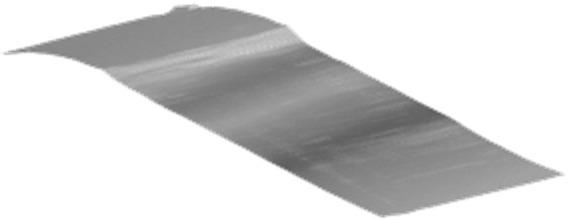
Surface with rutting: four samples Jiasong Highway	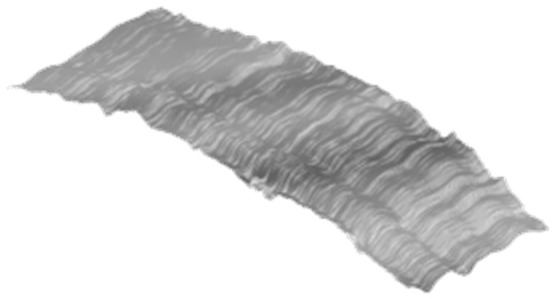
Rough surface: three samples Lianqun Highway	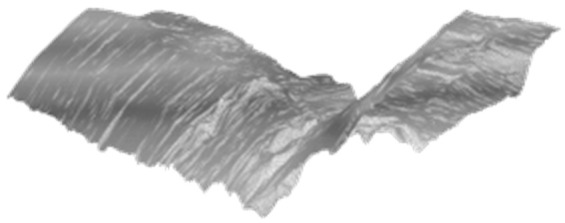

**Table 4:** Gallaway model and USF model.

Gallaway model	$v_{p}=0.9143\cdot SD^{0.04}P_{t}^{0.3}{\left(TD+0.794\right)}^{0.06}A$ $A=\max.of\{\begin{array}{l} \left(\frac{12.639}{WFT^{0.06}}\right)+3.507\\ \left[\left(\frac{22.351}{WFT^{0.06}}\right)-4.97\right]MTD^{0.14} \end{array}$
USF model	$v_{p}=W^{0.2}P_{t}^{0.5}\left(\frac{0.82}{WFT^{0.06}}+0.49\right)$

**Table 5:** Variables for hydroplaning speed estimation.

Variable	Value
MTD	1.0 mm
Tire pressure (*P_t_*)	250 Kpa
Wheel load (*W*)	5000 N
SD	1.0
Tire tread depth	1.0 mm

## Data Availability

The data presented in this study are available on request from the corresponding author.
